# Propagation characteristics analysis of high-frequency radio waves in the lower ionosphere layers

**DOI:** 10.1016/j.heliyon.2024.e40963

**Published:** 2024-12-06

**Authors:** Atefeh Esmaeili-Karnawah, Reza Fallah, Seyed Mohammad Khorashadizadeh, Ali Reza Niknam

**Affiliations:** a Department of Physics, University of Birjand, Birjand, 9717434765, Iran; b Laser and Plasma Research Institute, Shahid Beheshti University, Tehran, 1983969411, Iran

**Keywords:** Lower ionosphere, Electromagnetic waves, Transmission coefficient, Propagation, Absorption coefficient, Earth's magnetic field

## Abstract

In this article, the propagation of high-frequency (HF) plane electromagnetic waves through the lower ionosphere is numerically investigated using the real geometry of the Earth's magnetic field in the northern hemisphere. For this purpose, the profiles of electron density and the collision frequency in the layers of the lower ionosphere (D- and E-region) are considered using the reported experimental data for day and night. The reflection, transmission, and absorption coefficients of HF radio waves in the frequency range of 3 to 30 MHz are calculated in the ionosphere plasma. The influences of the collision frequency profile, the magnetic dip angle of Earth's magnetic field, and left- and right-handed polarization on the propagation of HF radio waves have also been investigated. The obtained results indicate that the left-handed polarized wave propagates more easily through the lower ionosphere than the right-handed polarized wave because its transmission coefficient is larger during the day and night. The effect of polarization on the absorption and transmission coefficients is observed for wave frequencies less than 10 MHz at night. It is also demonstrated that the effect of magnetic dip angle on the propagation of left- and right-handed polarized waves exhibits opposite trends in the lower ionosphere. The results show that the propagation of radio waves in the frequency range of 3 to 30 MHz is easier during the night, and the linear decreasing profile of the collision frequency compared to the fixed collision frequency increases the wave transmission in the lower ionosphere.

## 1 Introduction

The interaction of electromagnetic (EM) waves with ionosphere plasma is intriguing in plasma physics. The physics of the ionosphere is related to the physics of plasma since the ionosphere medium acts as weakly ionized natural plasma in which there is a considerable density of ions and free electrons. It can be called a natural plasma laboratory. The ionosphere is a low-ionized plasma that can interact with an electromagnetic wave [1], [2], [3], [4]. Given that the ionosphere can significantly affect the propagation of radio waves, many communication systems use the ionosphere to reflect radio signals over long distances [5], [6], [7], [8]. The amount of ionization within the ionosphere changes with time and shifts by day, year, and numerous other outside impacts. The Sun's radiation, which causes ionization, is only obvious during the day, and it is one of the most common reasons for the alteration in electron density. Whereas the sun's radiation causes atoms and molecules to be separated into free electrons and positive ions, the inverse effect also happens [9]. When a negative electron collides with a positive ion, they are pulled into each other and may combine. In this manner, the two inverse effects of division and recombination occur [10]. This is known as the state of dynamic equilibrium. Accordingly, the amount of ionization depends on the rate of ionization and recombination, and it encompasses a notable impact on radio communications [11].

The role of the ionosphere in the propagation of EM waves depends on the type of transmitted wave and geographical location. Ground waves are persistently in contact with the Earth's surface and don't make use of reflection from the ionosphere. Sky wave propagation permits transmitted signals to be reflected off a part of the ionosphere. The propagation of sky waves is also utilized to communicate over long distances [12], [13], [14]. The ionosphere is a region that changes due to reasons such as the time of day, the position of the sun and its radiation, and the geographic region of the world. As a result, radio communication using the ionosphere changes from one day to another and also from one hour to another [15]. The ionosphere is broadly known for influencing signals on the short-wave radio bands where it reflects signals empowering these radio communications signals to be heard over wide distances. Radio stations have used for a long time, the ionosphere properties to empower them to supply radio communications coverage around the world. Even though not as dependable as satellite communications, it isn't approximately as costly and can be a great protector in case satellite communications fail [16], [17], [18]. Furthermore, high-frequency (HF) wave propagation is broadly utilized for numerous organizations that require long-distance communication. As a result, HF wave propagation utilizing the ionosphere is likely to be utilized indefinitely as a form of radio communication. The study of how radio communications will be conceivable and how radio signals may propagate is of extraordinary intrigue to an assortment of radio communications users ranging from broadcasters to radio beginners and two-way radio. Electromagnetic wave propagation in the plasma will be influenced by the modern dynamical effect of Larmor gyration. Ruck et al. and Staelin present the electromagnetic wave reflection from dielectric slab by 2×2 matrix approaches [19], [20]. Vidmar studied the plasma layer at atmosphere pressure and found that this type of plasma can be used as a broadband absorber [21]. The reflection, absorption, and transmission of microwave waves in a non-uniform magnetic plasma slab have been examined by Larussy and Roth [22]. Guo and Wang studied the effect of negative ions on the absorption in atmosphere discharge uniform plasma layer [23]. The interaction of electromagnetic waves with the plasma slab with non-uniform electron density distribution has been investigated by different numerical methods. To investigate the transmission and absorption of EM waves in non-uniform plasma, various algorithms such as the scattering matrix method (SMM) [24], [25], the finite-difference time-domain (FDTD) method [26], the Wentzel-Kramers-Brillouin (WKB) method [27], and the integro-differential equation method [28] have been proposed.

The ionosphere as the layer of the Earth's atmosphere, which is created by solar photo-ionization and solar X-ray radiation, is located at an altitude of 60 to 700 km above the Earth's surface and the ionosphere regarding the electron density is divided into three regions: D, E and F [5]. Due to the importance of the ionosphere in the propagation of radio waves, in this research, we investigate the propagation of left-hand circular polarized (LHCP) and right-hand circular polarized (RHCP) plane waves in the lower ionosphere and influences of electron density and collision frequency profiles, magnetic dip angle of the Earth's magnetic field and also the polarization on the power of transmission, reflection, and absorption are numerically studied during day and night. This article is organized as follows: in Sec. 2, the theoretical framework, specifications, and basic formulas are introduced, Sec. 3 is dedicated to stating the results and discussion, and Sec. 4 concludes the paper with a summary.

## 2 Theoretical considerations

**Figure 1:**
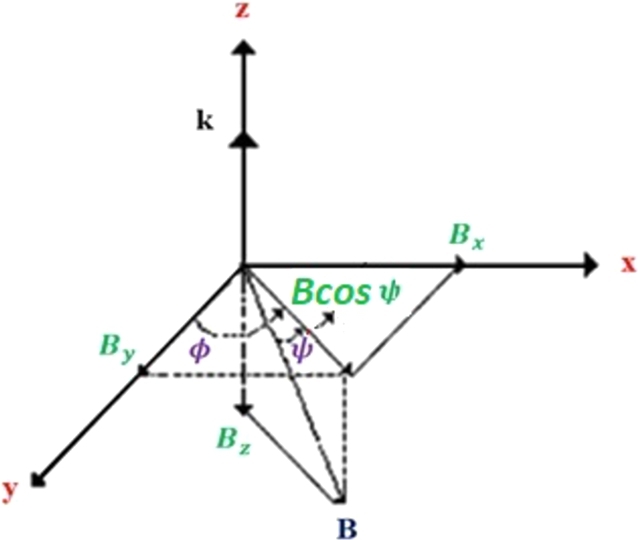
The scheme of the Earth's magnetic field for the northern hemisphere.

It is assumed that the radio waves in the range of frequencies 3 to 30 MHz, collide with Earth's ionosphere perpendicularly. The electric field of plane EM wave along z-direction can be expressed as

(1) $$\overset{\to }{E}=\overset{\to }{E_{\circ }}e^{\left(i\omega t-\overset{˜}{\gamma }z\right)},$$

where *ω* and $\overset{˜}{\gamma }$ are the EM wave frequency and the complex dielectric function, respectively, defined as

(2) $$\overset{˜}{\gamma }=i\frac{\omega }{c}\sqrt{\overset{˜}{\varepsilon }}=\alpha +i\beta ,$$

where *α* and *β* are known as the attenuation and phase coefficients, respectively, and $\overset{˜}{\varepsilon }$ is the complex permittivity of the ionosphere plasma. It is assumed that the z-axis of the coordinate system with its origin located on the ground is vertical upwards. The x- and y-axis represent geographic eastward and northward in the northern hemisphere, respectively. In Fig. 1, the scheme of the geomagnetic field (*B*) in the northern hemisphere is shown. In this figure, *ψ* is the angle that the Earth's magnetic field makes with the xy-plane in the northern hemisphere (magnetic dip angle), and *ϕ* is the angle between the y-axis and Bcosψ (magnetic declination angle).

The ionosphere is a weakly ionized, non-uniform and collisional plasma. In this work, in the presence of the Earth's magnetic field in the northern hemisphere, the ionosphere plasma is modeled as a series of magnetized uniform plasma slabs, in which the EM wave being absorbed and transmitted in each slab and reflected at the interface of each slab. Furthermore, the complex permittivity of magnetized plasma, when the EM wave propagating through a homogeneous plasma slab is expressed as [29]

(3) $${\overset{˜}{\varepsilon }}_{r}=1-\frac{\left(\frac{\omega_{P}^{2}}{\omega^{2}}\right)\left[1\pm \frac{\omega_{c}}{\omega }sin\psi \right]}{{\left[1\pm \frac{\omega_{c}}{\omega }sin\psi \right]}^{2}+{\left(\frac{\nu }{\omega }\right)}^{2}}-i\frac{\nu }{\omega }\frac{\left(\frac{\omega_{P}^{2}}{\omega^{2}}\right)}{{\left[1\pm \frac{\omega_{c}}{\omega }sin\psi \right]}^{2}+{\left(\frac{\nu }{\omega }\right)}^{2}}.$$

**Figure 2:**
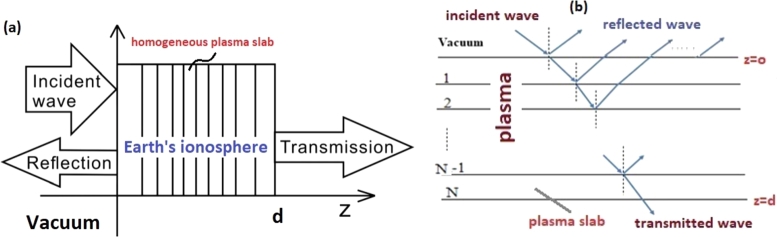
The schematic diagram of (a) electromagnetic wave propagation through ionosphere (b) wave absorption, transmission, and reflection at an arbitrary angle of incidence.

Here, ± sign indicates the left- and right-handed polarization, $\omega_{p}=\sqrt{\frac{n_{e}e^{2}}{m_{e}\varepsilon_{\circ }}}$ is the electron plasma frequency, $\omega_{c}=\frac{eB}{m_{e}}$ is the electron gyro-frequency and *ν* is the collision frequency. We divide the thickness of the ionosphere into N uniform plasma slabs. Accordingly, the schematic diagram of wave propagation characteristics through ionosphere, that its plasma is divided into a series of uniform plasma slab layers is depicted in Fig. 2(a). Also in Fig. 2(b), the absorption, transmission, and reflection of EM wave are shown at an arbitrary angle of incidence. In each slab, the plasma density and collision frequency are assumed to be uniform and the incident wave travels from one slab to the next by reflection at the interface. It is considered that the reflected wave is partially absorbed by the plasma slabs before it is reflected back out of the plasma. Therefore, the total reflected power can be calculated by neglecting multiple reflections between slab interfaces.

It is noteworthy that the complex permittivity of (j+1)th plasma slab ${\overset{˜}{\varepsilon }}_{r}(j+1)$ can be represented from Eq. (3), so the reflection constant of slab layer j+1 can be expressed as [30]

(4) $$\Gamma \left(j+1\right)=\frac{\sqrt{{\overset{˜}{\varepsilon }}_{r}\left(j\right)}-\sqrt{{\overset{˜}{\varepsilon }}_{r}\left(j+1\right)}}{\sqrt{{\overset{˜}{\varepsilon }}_{r}\left(j\right)}+\sqrt{{\overset{˜}{\varepsilon }}_{r}\left(j+1\right)}}.$$

The total incident power, reflected power, transmitted power, and absorbed power are denoted by $\;P_{i},\;P_{r},\;P_{t}$ and $\;P_{a}$, respectively, which $P_{i}=P_{r}+P_{t}+P_{a}$. If the reflection constant of each layer is calculated, then the total reflection and transmission coefficients can be obtained as follows [30]:

(5) $$R=\frac{P_{r}}{P_{i}}=\left\{\;{\left|\Gamma \left(1\right)\right|}^{2}+\sum_{n=2}^{N}\left({\left|\Gamma \left(n\right)\right|}^{2}\times \prod_{j=1}^{N-1}\left(exp\left[-4\alpha \left(j\right)d(j)\right]\left(1-{\left|\Gamma \left(j\right)\right|}^{2}\right)\right)\right)\;\;\right\}\;\;,$$

(6) $$T=\frac{P_{t}}{P_{i}}=\prod_{j=1}^{N}\left(exp\left[-2\alpha \left(j\right)d(j)\right]\left(1-{\left|\Gamma \left(j\right)\right|}^{2}\right)\right)\;,$$

where d (j) is the width of the j-th slab and N is the total number of slabs. Furthermore, the total absorption coefficient is calculated by A=1−R−T.

## 3 Results and discussion

In this section, to better understand the propagation of radio frequency waves in the ionosphere plasma under the Earth's magnetic field, we discuss the effects of collision frequency, EM wave polarization, orientation of the Earth's magnetic field and propagation time on the transmission, reflection and absorption coefficients in the ionosphere layers. For this purpose, the plasma density profiles are obtained for the lower layers (D and E) of ionosphere during daytime from the data reported in reference [31] as follows:

(7) $$n_{1}(z)=2\times 10^{4}(z-60\times 10^{3})\;+5\times 10^{7}\;\;\left(m^{-3}\right)\;\;\;\;\;\;\;\;\;\;\;\;\;60\;km≺\;z\;≺65\;km,$$

(8) $$n_{2}(z)=1.5\times 10^{4}(z-60\times 10^{3})+7.5\times 10^{7}\left(m^{-3}\right)\;\;\;\;\;\;\;\;\;\;\;\;\;\;\;65\;km≺\;z\;≺75\;km,$$

(9) $$n_{3}(z)=2.85\times 10^{6}(z-60\times 10^{3})-4.24\times 10^{10}\left(m^{-3}\right)\;\;\;\;\;\;\;\;\;\;\;\;\;\;\;75\;km≺\;z\;≺110\;km,$$

In addition to the electron density, the ionosphere collision frequency profile is also inhomogeneous during the daytime, and based on the data reported in reference [31] can be approximated as

(10) $$\nu (z)≃-400\;(z-60\times 10^{3})\;+2\times 10^{7}\;\;\;\;\;(Hz)\;\;\;\;\;\;\;\;\;\;\;\;\;\;\;\;\;\;\;\;\;60\;km≺\;z\;≺110\;km.$$

On the other hand, the density and collision frequency profiles of the lower ionosphere during night-time are different from those during day-time and can be obtained as follows [31]:

(11) $$n(z)=\;\;133\;\times 10^{3}(z-95\times 10^{3})\;+25\times 10^{6}\;\;\;\;\;(m^{-3})\;\;\;\;\;\;\;\;\;\;\;\;\;95\;km≺\;z≺110\;km,$$

(12) $$\nu (z)\overset{˜}{=}-6(z-95\times 10^{3})+10^{5}\;\;\;\;\left(Hz\right)\;\;\;\;\;\;\;\;\;\;\;\;\;\;\;\;\;\;\;\;\;\;\;95\;km≺\;z≺110\;km.$$

**Figure 3:**
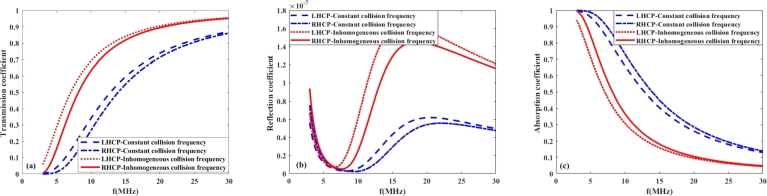
(a) Transmission (b) Reflection (c) Absorption coefficient versus wave frequency in the lower ionosphere at day-time for *B* = 0.4*G*, *ψ* = 30^*o*^.

**Figure 4:**
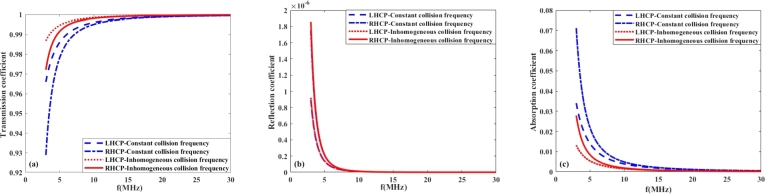
(a) Transmission (b) Reflection (c) Absorption coefficient versus wave frequency in the lower ionosphere at night-time for *B* = 0.4*G*, *ψ* = 30^*o*^.

However, due to the slow variation of the collision frequency with distance z during both day and night, it is reasonable to consider the collision frequency as a constant parameter in the study of wave propagation in the lower ionosphere layers. Furthermore, the polarization of high frequency radio waves, specifically LHCP and RHCP, significantly impacts their transmission, absorption, and reflection coefficients in the lower ionosphere, which in turn affects radio communication. During transmission, the ionosphere can preferentially transmit one polarization over the other, depending on its electron density and the angle of incidence. Absorption rates also vary with polarization, as the ionosphere's free electrons interact differently with LH and RH waves, potentially leading to reduced signal strength for one polarization. Additionally, these effects are particularly pronounced during different times; for instance, increased ionization during the day can lead to higher absorption rates, limiting communication range, while nighttime conditions, with reduced ionization, can enhance wave transmission and improve communication quality. Therefore, understanding the impact of wave polarization is crucial for optimizing high frequency radio communication under varying ionospheric conditions. On this basis, Figure 3, Figure 4(a-c) illustrate the coefficients of transmission, reflection, and absorption of LHCP and RHCP waves for constant and inhomogeneous collision frequency at day and night time, when B=0.4G. The EM wave frequency is taken from 3 to 30 MHz and constant collision frequency is chosen $2\times 10^{7}Hz$. Fig. 3(b) shows that by increasing the incident wave frequency from 3 to 30 MHz per day, the reflection coefficient remains very small and first decreases, then increases, and finally decreases again. The reflection coefficient is calculated from Eq. (5), which depends on complex permittivity and wave frequency, thus the maximum in the reflection coefficient can be attributed to several factors, including the specific frequency of the incident wave and the electron density profile of the ionospheric layers. Moreover, the results of Fig. 3(a-c) demonstrate that by increasing the wave frequency, the transmission and absorption coefficients are increased and decreased, respectively, and it can be seen that for the inhomogeneous collision frequency case, the reflection and transmission coefficients are greater than the homogeneous case. Fig. 4(b) shows that at night-time, the inhomogeneity of the collision frequency has not affected the reflection coefficient due to its small value and has caused a decrease in the absorption power rate compared to the case of a constant collision frequency. It is followed by debt that has increased transmission power. Comparing the results of Figure 3, Figure 4(a-c) reveals that the absorption of waves in the lower ionosphere is greatest during local day, when the ionospheric densities are highest. So the propagation of waves in the lower ionosphere is better at night than during daytime. Moreover, the obtained results indicate that during the day and night-time, the RHCP wave more strongly absorbed than LHCP ones. From Eq. (3), one can see that the complex permittivity and consequently, the wave number depends on the polarization of electromagnetic waves. The RHCP wave drives electrons in the direction of their cyclotron motion, while the LHCP wave has a rotation sense opposite to the cyclotron motion of the electrons. Given that the refractive index and the absorption coefficient are respectively proportional with the real and imaginary parts of the wave number, therefore, left-handed and right-handed circularly polarized waves impact their transmission, absorption, and reflection coefficients in the lower ionosphere, which in turn affects HF radio communication.

**Figure 5:**
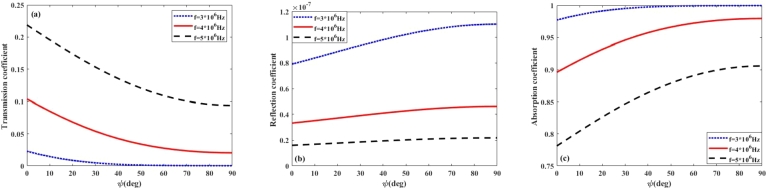
(a) Transmission (b) Reflection (c) Absorption coefficient of RHCP wave versus wave frequency in the lower ionosphere at day-time for *B* = 0.4*G*.

**Figure 6:**
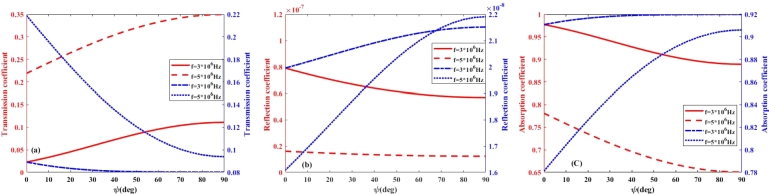
(a) Transmission (b) Reflection (c) Absorption coefficient versus wave frequency in the lower ionosphere at day-time for *B* = 0.4*G*, the right- and left-handed axes for RHCP and LHCP, respectively.

**Figure 7:**
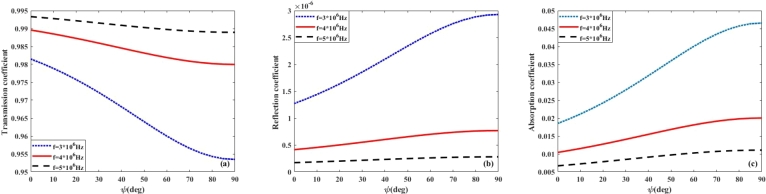
(a) Transmission (b) Reflection (c) Absorption coefficient of RHCP wave versus wave frequency in the lower ionosphere at night-time for *B* = 0.4*G*.

**Figure 8:**
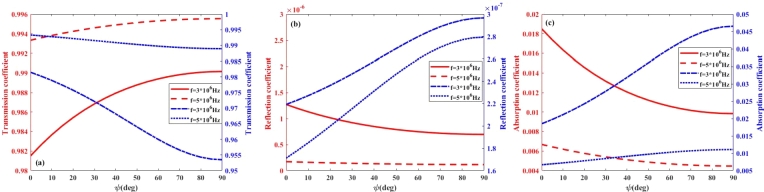
(a) Transmission (b) Reflection (c) Absorption coefficient versus wave frequency in the lower ionosphere at night-time for *B* = 0.4*G*, the right- and left-handed axes for the RHCP and LHCP, respectively.

On the basis on Eq. (3), it is expected that the absorption, transmission, and reflection coefficients, will depend on the magnetic dip angle of the Earth's magnetic field. This magnetic dip angle significantly influences the propagation of high-frequency (HF) radio waves, particularly in relation to their absorption and transmission coefficients within the lower ionosphere. As HF waves interact with the ionosphere, the dip angle modifies the orientation of the waves relative to the magnetic field, thereby affecting their propagation characteristics. Typically, a steeper dip angle results in increased absorption of RHCP waves, as the interaction with the ionospheric plasma becomes more pronounced, especially at lower frequencies. The rate of absorption is also influenced by the polarization of the waves; for example, left-handed and right-handed polarized waves may experience varying levels of absorption depending on their alignment with the magnetic field. Accordingly, in Figure 5, Figure 6, Figure 7, Figure 8(a-c), the effect of magnetic dip angle on the coefficients of transmission, reflection, and absorption of left- and right-hand polarized radio waves is shown for day and night, respectively. From these figures, it can be seen that for RHCP wave and in the frequency range of 3 to 5 MHz, by increasing the magnetic dip angle (*ψ*) from 0 to $90^{\circ }$, the transmission and absorption coefficients significantly decrease and increase, respectively. While it is observed that for the LHCP wave in the frequency range of less than 5 MHz, with the increase of *ψ*-angle, the transmission coefficient increases, and the absorption coefficient decreases considerably. Also, from Figure 5, Figure 6(a-c), it can be seen that by increasing the wave frequency, the impact of magnetic dip angle on the transmission and absorption coefficients increases, also for LHCP wave, the distribution of these curves is opposite to that obtained for RHCP wave in the daytime. It is expected that the similar results will be obtained in the investigation of the impact of polarization and *ψ*-angle on the wave propagation at night-time. As Figure 7, Figure 8(a-c) show that the changes of transmission and absorption coefficients with increasing the wave frequency and magnetic dip angle (*ψ*) at night, are similar to those during daytime. The result is that during the daytime, solar radiation increases the ionization in both the D and E layers, leading to higher electron densities. This enhanced ionization results in increased absorption of electromagnetic waves, particularly at lower frequencies, as the waves interact more intensely with the free electrons present in these layers. Consequently, the transmission and reflection coefficients may be lower during the day, as more energy is absorbed. In contrast, at night, the ionization levels decrease, leading to reduced absorption and improved transmission coefficient, allowing for better transmission of signals over longer distances. The collision frequency profile plays a crucial role in these differences. During the day, the collision frequency is generally higher due to the increased number of electrons, which enhances scattering and absorption processes. At night, the lower collision frequency allows for more efficient transmission of electromagnetic waves, as the reduced interactions with the ionospheric plasma facilitate clearer signal propagation. Thus, understanding these dynamics is essential for optimizing communication strategies that rely on HF radio waves, as the effectiveness of transmission can vary significantly based on the time of day and the specific characteristics of the ionospheric layers.

## 4 Conclusions

The propagation of plane radio waves in the lower ionosphere layers in the presence of the Earth's magnetic field was investigated during the day and night. The electron density and the collision frequency in the ionosphere layers vary between day and night, so the distribution of these quantities was obtained from the reported experimental results. This study examines the influence of magnetic dip angle of the Earth's magnetic field, homogeneous and inhomogeneous collision frequency as well as day and night conditions on the reflection, transmission, and absorption coefficients for the LHCP and RHCP waves. From the obtained numerical results, it was observed that the propagation of HF waves is sensitive to the collision frequency and time of day and night. The polarization of the waves also plays a critical role; left-handed and right-handed circularly polarized waves can interact differently with the ionospheric plasma, affecting their transmission and reflection coefficients. It was shown that more than 80% of the RHCP wave power with a frequency of less than 5 MHz can be absorbed in the lower ionosphere during the day, and this amount of absorption decreases at the night, due to decreasing of electron density and collision frequency, consequently the transmission of radio wave becomes better obviously. In addition, it was observed that the high electron density in the ionosphere layers during the day can reduce the transmission coefficient, while it can increase the bandwidth of radio wave absorption. It was also found that the impact of the inhomogeneity of the collision frequency causes the absorption coefficient to decrease compared to the case of the homogeneous collision frequency. In addition, higher frequency waves tend to penetrate deeper into the ionosphere, allowing for longer-range communication, but may experience higher absorption rates due to increased ionization, while lower frequency waves are more likely to be reflected back to Earth. Understanding these dynamics is essential for optimizing HF communication systems, particularly in applications such as satellite communication, remote sensing, geographic information systems (GIS), also aviation, maritime, and emergency communications, where reliable long-distance transmission is crucial.

## CRediT authorship contribution statement

**Atefeh Esmaeili-Karnawah:** Writing – original draft, Methodology, Formal analysis, Data curation. **Reza Fallah:** Writing – review & editing, Validation, Methodology, Investigation, Conceptualization. **Seyed Mohammad Khorashadizadeh:** Writing – review & editing. **Ali Reza Niknam:** Writing – review & editing, Validation, Investigation, Conceptualization.

## Declaration of Competing Interest

The authors declare that they have no known competing financial interests or personal relationships that could have appeared to influence the work reported in this paper.

## Data Availability

Data will be made available on request.
